# Adrenocortical Sarcomatoid Carcinoma Revealed by an Adrenal Incidentaloma: A Case Report

**DOI:** 10.7759/cureus.53720

**Published:** 2024-02-06

**Authors:** Samia Bentaleb, Ghita Bourkadi, Hayat Aynaou, Houda Salhi

**Affiliations:** 1 Endocrinology, Diabetes and Metabolism, Centre Hospitalier Universitaire Hassan II, Fes, MAR

**Keywords:** incidentaloma, histology, adrenal gland, sarcomatoid, carcinoma

## Abstract

Sarcomatoid carcinoma of the adrenal gland represents an exceedingly unusual and highly aggressive form of adrenocortical carcinoma. Its diagnosis is challenging because of its dual histological components: epithelial and sarcomatoid. Most patients are diagnosed at a late stage and die within months of diagnosis. We report on a 51-year-old man who had adrenocortical sarcomatoid cancer. It was diagnosed as a unilateral left adrenal incidentaloma discovered on a CT scan carried out for abdominal pain. By means of this case, we will present the clinical, radiological, and histological profile of this tumor.

## Introduction

Adrenocortical sarcomatoid carcinoma (ASC) is a biphasic malignant tumor with both an epithelial and a sarcomatoid component. Compared to other sites such as the lung, kidney, breast, and esophagus, adrenal localization is infrequent, with an incidence of two cases for every million people [1,2]. The earliest documented case of this disease dates back to 1989 when Collina and colleagues reported it [3]. To the best of our present awareness, a total of 22 cases are currently recorded in the literature at present [4]. Depending on whether symptoms related to excessive hormone secretion are present or absent, ASC can manifest as either a functional or non-functional tumor [2]. Its definitive diagnosis is histological and immunohistochemical. There are no standardized norms for treating this cancer, owing to the small number of cases reported in the literature. Surgical intervention is the first choice. It is considered a high-risk metastatic disease with a poor prognosis [5]. We report a case of sarcomatoid carcinoma of the left adrenal gland with liver and lung metastases discovered as a unilateral adrenal incidentaloma.

## Case presentation

**Figure 1:**
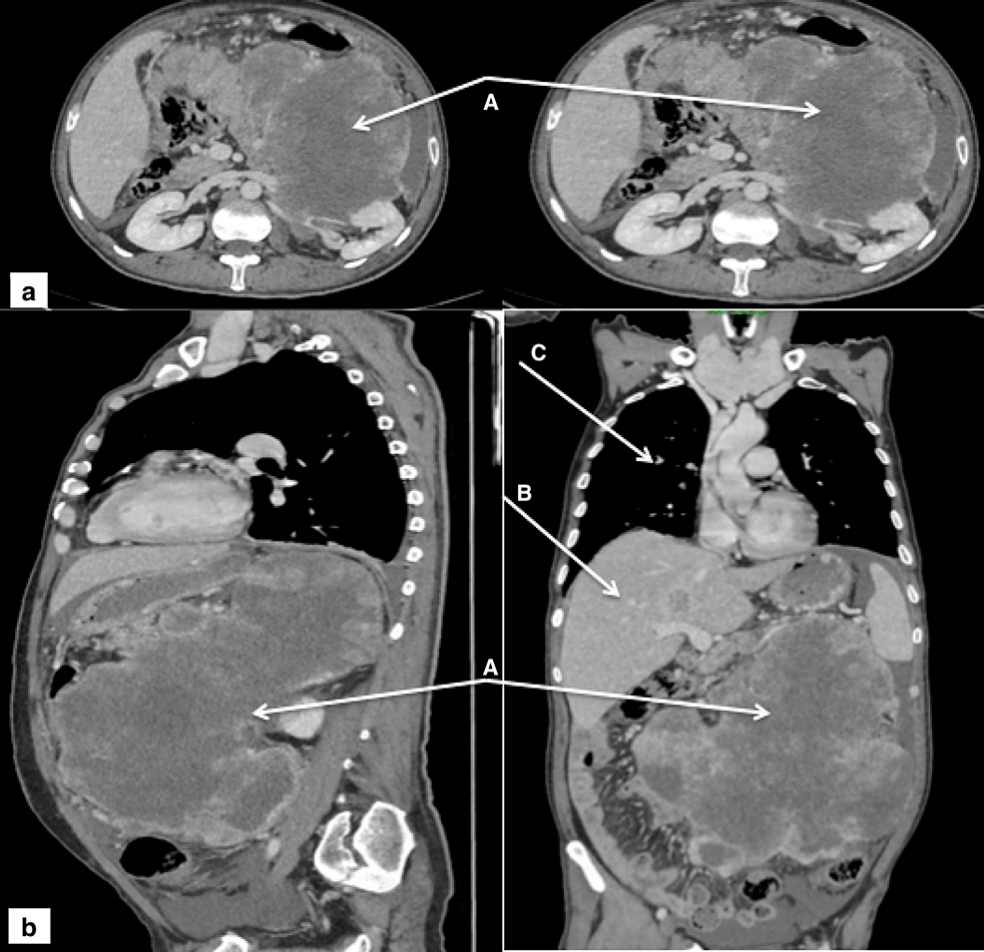
Thoracoabdominal CT scan: (a) cross-section and (b) coronal section. A: Left adrenal mass measured 13 x 16 x 24 cm, massively necrotic. B: Secondary hepatic localization. C: Secondary pulmonary localization.

A 51-year-old man with no previous medical history was directed to investigate a unilateral adrenal incidentaloma. It was revealed on an abdominal CT scan performed for diffuse abdominal pain. The patient complained of both asthenia and a significant weight loss of 17.6% over a seven-month period. On admission, the examination uncovered a large mass located in the left hypochondrium, firm and painful on palpation, without clinical signs of hypersecretion, especially the signs of Menards triad (headache, palpitations, and sweating) or Cushing's syndrome, and without signs of feminization. The CT scan showed a left adrenal mass with irregular contours, of a heterogenous density and a significant peripheral contrast enhancement, delineating large areas of central necrosis. The mass measured 13 x 16 x 24 cm in diameter and involved the pancreas, left kidney, spleen, and diaphragmatic dome. It also invaded the inferior vena cava and the inferior mesenteric vein and resulted in thrombosis of the left renal vein. Secondary localizations in the liver and the lungs were found (Figure 1).

The biological work-up showed an elevated midnight cortisol level, an increased free cortisol in the urine, and a pathological suppression of dexamethasone. The 24-hour urinary catecholamines, dehydroepiandrosterone sulfate, and estradiol levels were normal.

The case was reviewed at a multidisciplinary consultation meeting, and it was concluded that due to the advanced and metastatic nature of the mass, it was not practical to remove it by surgery. Biopsy was therefore recommended. The patient benefited from an ultrasound-guided liver biopsy, but the results were inconclusive. After a multidisciplinary decision, a second biopsy of the adrenal mass was performed. Histological and immunohistochemical studies revealed a spindle-shaped malignant proliferation without MDM2 amplification, which is compatible with adrenal carcinoma in its sarcomatoid variant with secondary hepatic localization.

The patient was treated with anticoagulants for splenic thrombosis and analgesics for pain and was directed to the department of oncology for management. Unfortunately, he died a week later.

## Discussion

Based on the available information, we present the 23rd case of sarcomatoid carcinoma of the adrenal cortex [4]. The median age at presentation of ASC is 54.3 years, with a gender ratio (female to male) of 1.4 [6]. However, our patient was a 51-year-old man.

As in our case, these tumors can be detected by non-specific symptoms, such as abdominal pain, abdominal mass, asthenia, and weight loss. If they are functional, they can manifest themselves with symptoms such as Cushing's syndrome, high blood pressure, feminization, or masculinization [7].

Diagnosing ASC before surgery remains challenging [8]. Adrenal biopsies are not commonly performed in potentially malignant adrenal masses. The conclusive diagnosis is usually established by surgical exploration [7]. This includes histological and immunohistochemical analysis [8]. In our case, the diagnosis was made after an anatomopathological study of the biopsy, as the mass was considered to be unresectable after a multidisciplinary review.

Carcinomas with a sarcomatous component consist mainly of malignant spindle cells lacking distinct heterologous differentiation, usually accompanied by regions featuring typical epithelial differentiation [2]. In our situation, a population of malignant spindle cells with no discernible differentiation was observed, suggesting an adrenal sarcomatoid adenocarcinoma.

Although this type of tumor represents a significant diagnostic challenge from a clinical, radiological, and histopathological point of view, its early detection will ensure a better long-term outcome in this aggressively treated cancer. It is difficult to reach an evidence-based consensus on the optimal oncological treatment due to its rarity. Previous cases reported in the literature have shown variable use of adjuvant therapy. In eight cases, no additional therapy was documented [6]. Collina et al. [3] and Fischler et al. [9] reported chemotherapy using etoposide and cisplatin in three additional cases, while Sturm and colleagues reported using cisplatin alone [2]. Additionally, Fischler et al. reported using mitotane in conjunction with chemotherapy [9]. Only one of the prior cases involved radiotherapy, but it was assumed to be palliative treatment [3]. Our patient died before having been treated.

Age, disease progression, and the extent of surgical resection are important factors for assessing the prognosis of ACS. Histopathological features such as tumor weight and dimensions, atypical mitoses, presence of necrosis, high nuclear pleomorphism, and reduced number of cells with clear cytoplasm as well as vascular and capsular invasion have a substantial impact on the disease’s outcome [2]. The majority of ASC patients experience three to 12 months of postoperative survival following surgical treatment; the longest postoperative survival without metastasis was 17 months [4].

We are of the opinion that the most favorable opportunity for achieving long-term survival in cases of ASC lies in early detection before metastatic spread, followed by a radical and complete excision, along with adjuvant external radiotherapy to prevent local recurrence of the disease [6]. There is potential for novel therapeutic targets related to epithelial-to-mesenchymal transition (EMT) markers and stem cell factors [4].

## Conclusions

We have described the clinical and histopathological features of this extremely rare form of cancer. Identification of this histological subtype is essential due to its poor prognosis, justifying the use of a suitable immunohistochemical panel. The management of these tumors requires aggressive treatment and a multidisciplinary approach involving a team of experienced specialists.

To date, there is no consensus on the optimal management of ASCs. It is therefore crucial to understand this rare disease and develop effective treatments to improve its poor prognosis.
